# The oxido-metabolic driver ATF4 enhances temozolamide chemo-resistance in human gliomas

**DOI:** 10.18632/oncotarget.17737

**Published:** 2017-05-09

**Authors:** Daishi Chen, Manfred Rauh, Michael Buchfelder, Ilker Y. Eyupoglu, Nicolai Savaskan

**Affiliations:** ^1^ Translational Cell Biology and Neurooncology laboratory at the Department of Neurosurgery, Universitätsklinikum Erlangen (UKER), Friedrich-Alexander University of Erlangen–Nürnberg (FAU), Erlangen, Germany; ^2^ Department of Otolaryngology–Head and Neck Surgery, Chinese PLA General Hospital, Beijing, China; ^3^ Department of Pediatrics and Adolescent Medicine, Universitätsklinikum Erlangen (UKER), Friedrich-Alexander University of Erlangen–Nürnberg (FAU), Erlangen, Germany; ^4^ BiMECON Ent., Berlin, Germany

**Keywords:** autophagy, ferroptosis, apoptosis, cell death, glutamate

## Abstract

Malignant gliomas are devastating neoplasia with limited curative treatment options. Temozolomide (TMZ, Temcat^®^, Temodal^®^ or Temodar^®^) is a first-line treatment for malignant gliomas but the development of drug resistance remains a major concern. Activating transcription factor 4 (ATF4) is a critical oxido-metabolic regulator in gliomas, and its role in the pathogenesis of TMZ-resistance remains elusive. We investigated the effect of TMZ on human glioma cells under conditions of enhanced ATF4 expression (ATF4^OE^) and ATF4 knock down (ATF4^KD^). We monitored cell survival, ATF4 mRNA expression of ATF4 and xCT (SLC7a11) regulation within human gliomas. TMZ treatment induces a transcriptional response with elevated expression of ATF4, xCT and Nrf2, as a sign of ER stress and toxic cell damage response. ATF4 overexpression (ATF4^OE^) fosters TMZ resistance in human gliomas and inhibits TMZ-induced autophagy. Conversely, ATF4 suppression by small interfering RNAs (ATF4^KD^) leads to increased TMZ susceptibility and autophagy in comparison to wild type gliomas. ATF4^OE^ gliomas show reduced cell cycle shift and apoptotic cell death, whereas ATF4^KD^ gliomas reveal higher susceptibility towards cell cycle rearrangements. Hence, the migration capacity of ATF4^OE^ glioma cells is almost not affected by TMZ treatment. In contrast, ATF4^KD^ gliomas show a migratory stop following TMZ application. Mechanistically, xCT elevation is a consequence of ATF4 activation and increased levels of xCT amplifies ATF4-induced TMZ resistance. Our data show that ATF4 operates as a chemo-resistance gene in gliomas, and the tumor promoting function of ATF4 is mainly determined by its transcriptional target xCT. Therefore, therapeutic inactivation of ATF4 can be a promising strategy to overcome chemo-resistance and promote drug efficacy in human gliomas.

## INTRODUCTION

Malignant gliomas (abbreviated as gliomas) are the most lethal primary brain tumors in children and adults [1]. Gliomas are characterized by their invasive growth properties and space occupying effects causing clinical emergency [1, 2, 3]. Moreover, recent advances in neurooncology uncovered cytotoxic features of malignant gliomas impacting on brain cell viability. These processes can affect brain functioning and thereby exacerbating the space occupying effects and neurological deficits and [10]. The median survival time from diagnosis is approximately 14 months [1, 4]. From those, glioblastomas (GBM; WHO grade IV) are hallmarked by features as uncontrolled cellular proliferation, diffuse infiltration, and resistance to apoptosis and chemotherapy. The current standard-of-care for GBM patients includes adjuvant temozolomide (TMZ) treatment as part of the current multimodal approach. This treatment strategy is currently the best clinical practice, however, conferring still a median survival time of only 14.6 months compared with 12.2 months for patients receiving only radiotherapy [5]. Although temozolomide (TMZ brand names Temodal^®^ in Europe and Temcad^®^ in the USA) offers some hope to GBM patients with increasing progression free and overall survival of few months, a best 5 year survival rate of only 9.8% is currently achieved [1].

TMZ is a small 194 Da lipophilic orally available molecule with DNA alkylating activity. Converted to methyltriazen-1-yl-imidazole-4-carboxamide, TMZ acts cytotoxic via mispair and futile mismatch repair loop leading to apoptosis and autophagic cell death [6, 7]. Evasion of cell death and development of redox stability is one of the hallmarks of cancers and promotes tumorigenesis as well as chemo-resistance. Apoptosis is a common form of programmed cell death that can be engaged via the intrinsic or extrinsic pathways. Recently, it has been shown that the glutamate cystine exchanger xCT (SLC7a11) appears to be essential in the process of chemo-resistance in some cancer cell types [8, 9]. Hence, since xCT plays a pivotal role in tumor microenvironment interactions, i.e. inducing of peritumoral neuronal cell death and perifocal edema [2, 10], there is a quest for understanding the regulation of xCT and inhibiting compounds for this transporter [11, 12].

ATF4 is a member of the CREB/ATF transcription factor family, is ubiquitously expressed in human organs and can be activated in response to various stress signals, including anoxia, hypoxia, endoplasmic reticulum stress, amino acid deprivation, and oxidative stress [13, 14]. The stress-induced expression of ATF4 causes adaptive responses in cells through regulating the expression of target genes involved in amino acid synthesis, differentiation, metastasis, angiogenesis, and drug resistance [15, 16, 17]. Moreover, increased expression of ATF4 has been reported to go along with the malignancy in human tumor pathologies [18, 19]. Thus, the upregulated expression of ATF4 is thought to facilitate tumor progression. Mechanistically, transcription of many essential genes involved in tumor cell proliferation are regulated by ATF4 [18, 20, 21]. However, there are various effector genes induced by ATF4 [22, 23], whereas it has not yet elucidated which of them are operational for malignant transformation, tumor progression and therapy resistance.

In this study, we investigated the temozolomide effects on human gliomas with concomitant ATF4 expression. Further, we analyzed candidate genes which drive ATF4-dependent chemotherapeutic resistance. In the present study we unraveled ATF4 as a key promoter for temozolomide resistance. Moreover, fostered ATF4 expression increases tumor migration which is almost not affected by TMZ treatment. Interestingly, the ATF4-induced chemo-resistance can be attenuated by xCT inhibition. Thus, our results indicate that ATF4 acts as a driver of cellular chemo-resistance via augmnented xCT expression.

## RESULTS

### TMZ up-regulates activating transcription factor 4 (ATF4) and xCT in glioma cells

**Figure 1:**
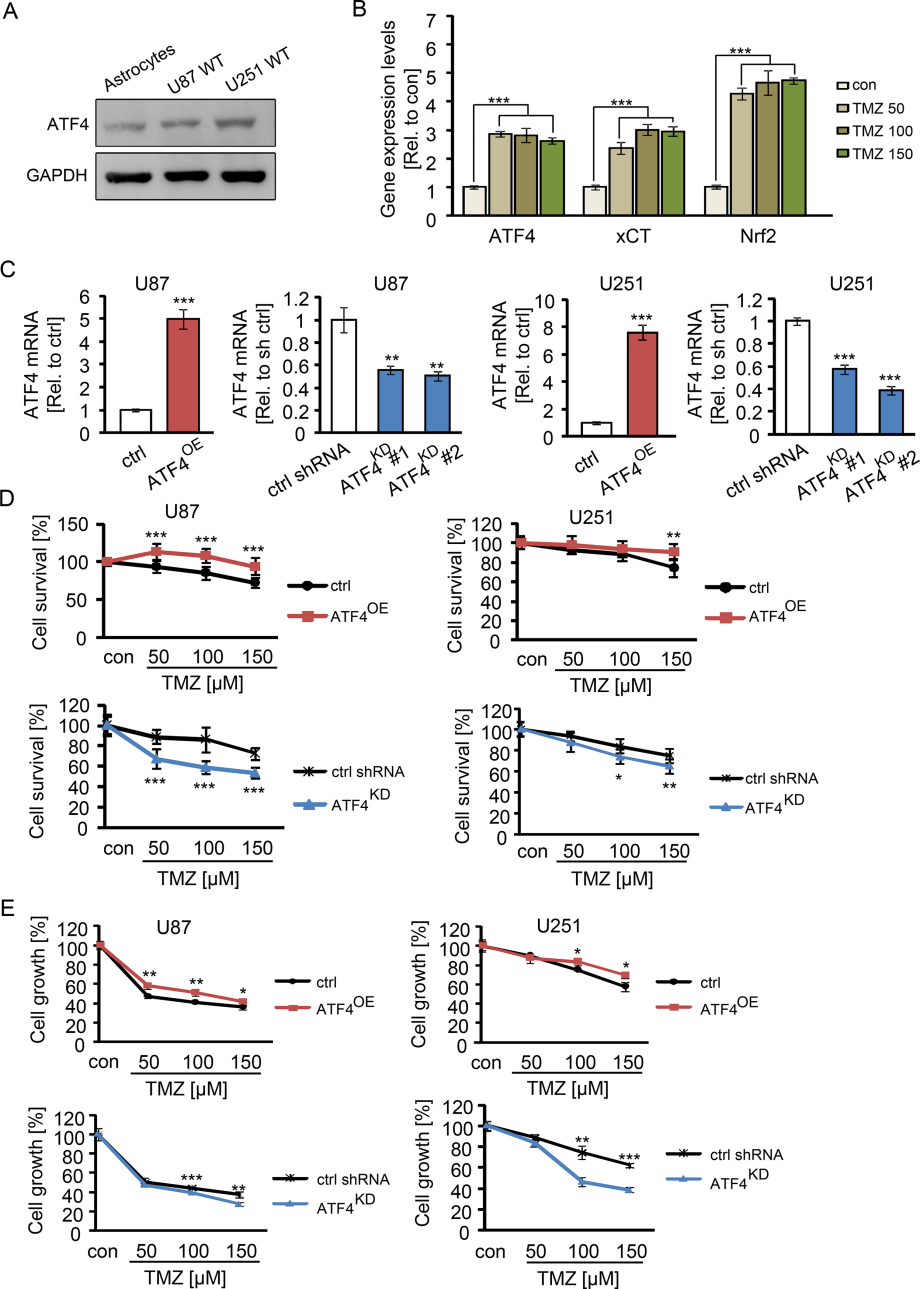
ATF4 induces TMZ resistance in glioma cells (**A**) Western blot analysis of ATF4 expression in human primary astrocytes, U87 WT (wild type) cells and U251 WT (wild type) cells. (**B**) U87 WT cells were subjected to TMZ in a series of concentrations as indicated for 48 h, then ATF4, Nrf2 and xCT mRNA levels were evaluated by RT-PCR. *n* = 3, ***P < 0.001 compared with con (untreated) using one-way ANOVA. CU87 and U251 cells were transfected with ATF4 cDNA and shRNAs as described in Material and methods. ATF4 mRNA was quantified by real time RT-PCR using the ΔΔCT method with GAPDH. D, ATF4^OE^ and ATF4^KD^ U87 and U251 cells were subjected to TMZ for 3 days in a series of concentrations as indicated. The cell survival was measured by MTT assay. E, After treatment with TMZ for 3 days, the total number of vital cells was monitored. *n* ≥ 8 per group. Statistical analysis was performed by unpaired Student's test, *P < 0.05, **P < 0.01, ***P < 0.001, ctrl (peGFP-N1) versus ATF4-GFP or ctrl shRNA versus ATF4 shRNA.

First, we investigated the expression levels of ATF4 in primary astrocytes and human glioma cell lines. Immunoblot analyses revealed that both human glioma cell lines U87 and U251 show increased ATF4 protein levels compared to non-transformed primary astrocytes (Figure 1A). To determine the contribution of ATF4 to the TMZ resistance of human gliomas, we next investigated the gene expression response following TMZ application. Increased ATF4, Nrf2 and xCT expression levels were detected in human glioma cells subsequently TMZ treatment for 48 h. The RT-PCR results revealed that TMZ at concentrations of 50 μM, 100 μM, and 150 μM markedly increased ATF4, Nrf2 and xCT mRNA levels (Figure 1B). These data indicate that TMZ increases ATF4 and xCT expression levels as part of the cellular stress response (Figure 1B).

### ATF4 expression levels correlate with TMZ resistance

To investigate the association between resistance to TMZ and ATF4 expression in glioma cells, we inhibited ATF4 expression by applying ATF4 specific shRNAs and created ATF4 overexpression by transfecting with vector containing ATF4 wildtype cDNA. We detected the expression levels of ATF4 in ATF4-modulated glioma cells by real time PCR (Figure 1C). ATF4-modulated U87 and U251 cells were seeded at a number of 3 × 10^3^ cells in 96-wells plate overnight prior drug application. Following the next day we treated cells with TMZ for 3 days at concentrations of 50 to 150 μM in order to investigate the correlation between ATF4 expression and TMZ sensitivity. The sensitivity of glioma cells to TMZ was significantly increased following ATF4 siRNA knockdown (Figure 1D, 1E). At 100 μM to 150 μM concentration of TMZ, 40% and 30% reduction in cell viability were observed in ATF4^KD^ U87 and ATF4^KD^ U251 cells, respectively. ATF4^OE^ cells were more resistant to TMZ compared to controls (Figure 1D, 1E). Noteworthy, cell proliferation of ATF4^OE^ cells was solely reduced at higher concentrations of TMZ (Figure 1E).

### Impact of ATF4 on TMZ-induced cell death

**Figure 2:**
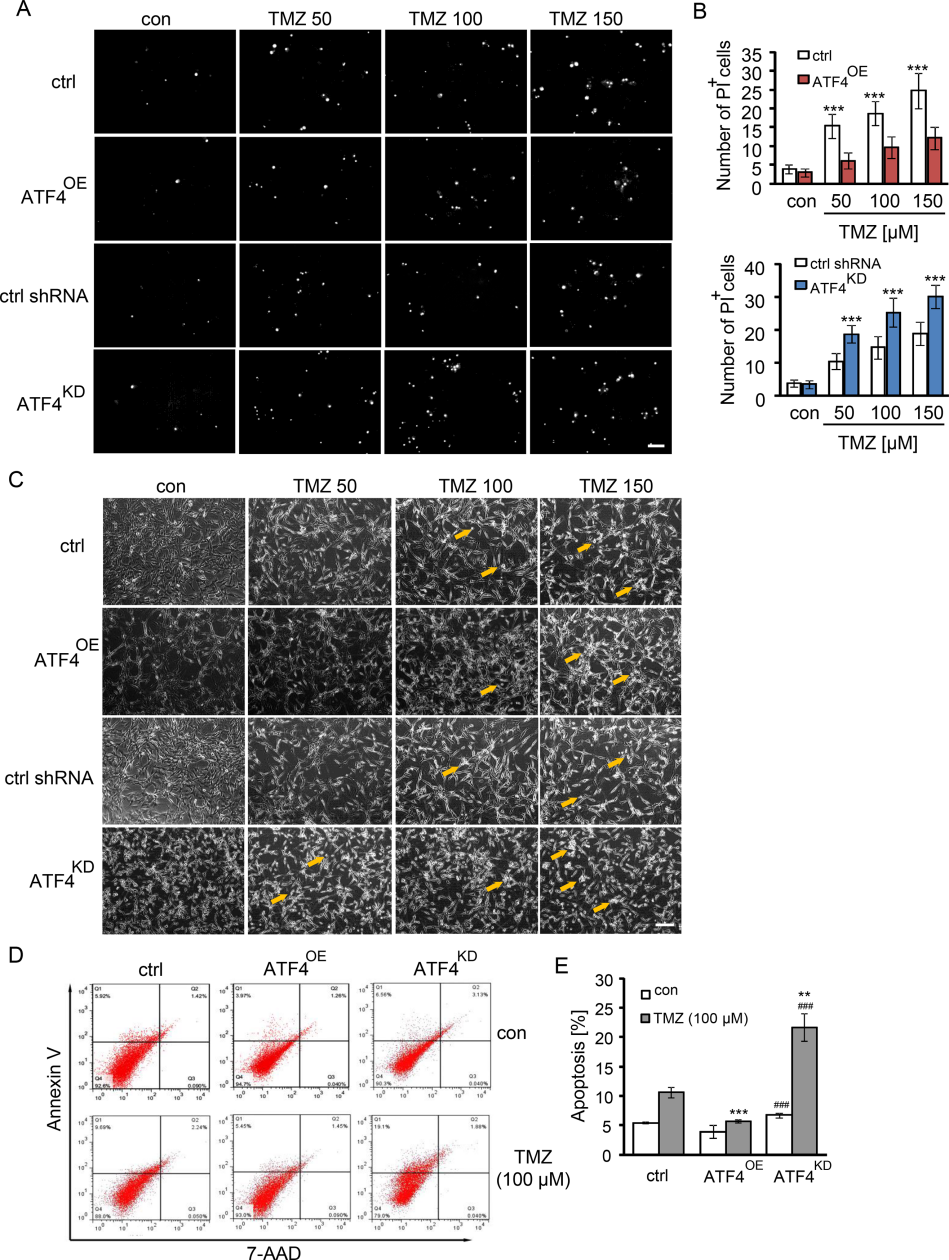
TMZ induces cell death in an ATF4-dependent manner (**A** and **B**) ATF4^OE^ and ATF4^KD^ U87 cells were treated with TMZ at indicated concentrations for 3 days. Cell death was demonstrated by propidium iodide (PI) staining. Scale bar represents 100 μm. *n* ≥ 3 per group. Statistical analysis was performed by unpaired Student's test. ***P < 0.001, ctrl (peGFP-N1) versus ATF4-GFP or ATF4 shRNA versus ctrl shRNA. (**C**) Visualization of ATF4^OE^ and ATF4^KD^ U87 cells treated with TMZ for 3 days. Scale bar represents 100 μm. (**D**) Cell death analysis was performed by flow cytometer with 7-AAD (late apoptosis) and Annexin V (early apoptosis) staining. (**E**) Quantification of apoptotic cell death in ATF4^OE^ and ATF4^KD^ U87 cells treated with TMZ. *n* = 3 per group. Statistical analysis was performed by unpaired Student's test. **P < 0.01, ***P < 0.001, ctrl versus ATF4-GFP or ctrl shRNA versus ATF4 shRNA; ^###^*P* < 0.001, ATF4-GFP versus ATF4 shRNA.

To observe whether ATF4 is responsible for TMZ-induced cell death in glioma cells, we analyzed cell death by propidium iodide (PI) staining after TMZ treatment. This cell death analysis demonstrated that dead cells increased with elevating TMZ dosage revealing significant differences between the ATF4^OE^ cells and ATF4^KD^ U87 cells (Figure 2A–2C). ATF4^KD^ U87 cells were more susceptible to TMZ compared to ATF4^OE^ U87 cells (Figure 2A, 2C). Moreover, clinically relevant concentrations of TMZ (100 μM) increased significantly the portion of apoptotic cells in ATF4^KD^ U87 cells compared with ATF4^OE^ cells, as assayed by flow cytometry with annexin V and 7-AAD double staining (Figure 2D, 2E).

**Figure 3:**
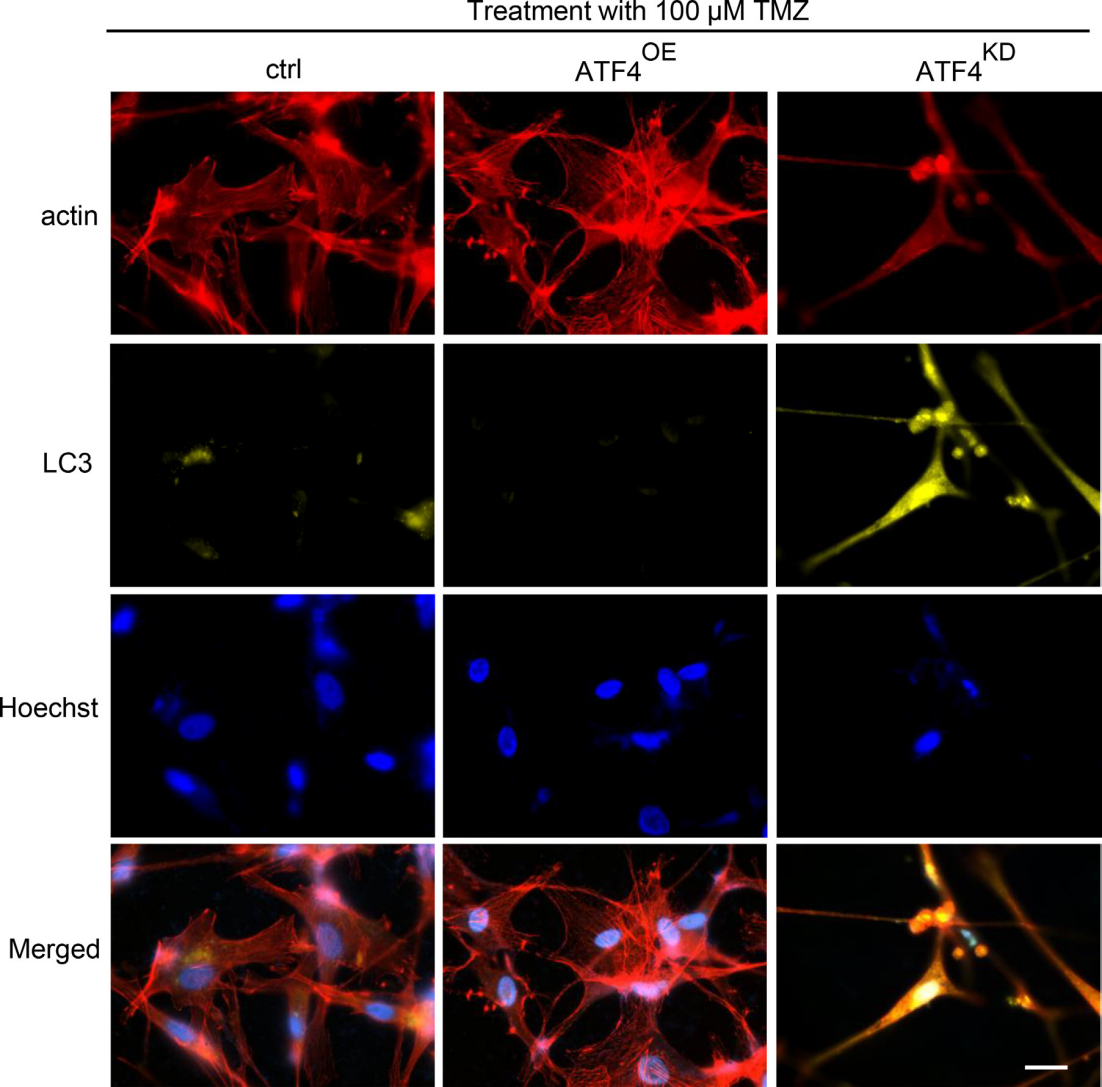
ATF4 counteracts TMZ-induced autophagy in glioma cells Representative images display actin (red), LC3 (yellow) and nuclei (blue). Autophagic vacuoles were monitored 72 h after 100 μM TMZ treatment. Increased autophagic signs were found in ATF^KD^ gliomas. Scale bar represents 50 μm.

We next facilitated the microtubule-associated protein 1 light-chain 3 (LC3) as a reliable marker for undergoing autophagic processes. Noteworthy, ATF4^KD^ U87 cells showed increased diffuse distribution of the microtubule-associated protein 1 light-chain 3 (LC3) puncta three days after TMZ treatment (Figure 3). In contrast, LC3 puncta were undetectable in ATF4^OE^ U87 cells, indicating ATF4 inhibits TMZ-induced autophagy in glioma cells (Figure 3). Additionally, ATF4^OE^ U87 cells display a polyplastic shape and are significantly bigger than ATF4^KD^ U87 cells. Conversely, ATF4^KD^ U87 cells are generally smaller and show a spindle-like phenotype with maximal two membrane extensions in contrast to controls (Figure 3).

### Effects of elevated ATF4 on TMZ-induced G2/M arrest in glioma cells

**Figure 4:**
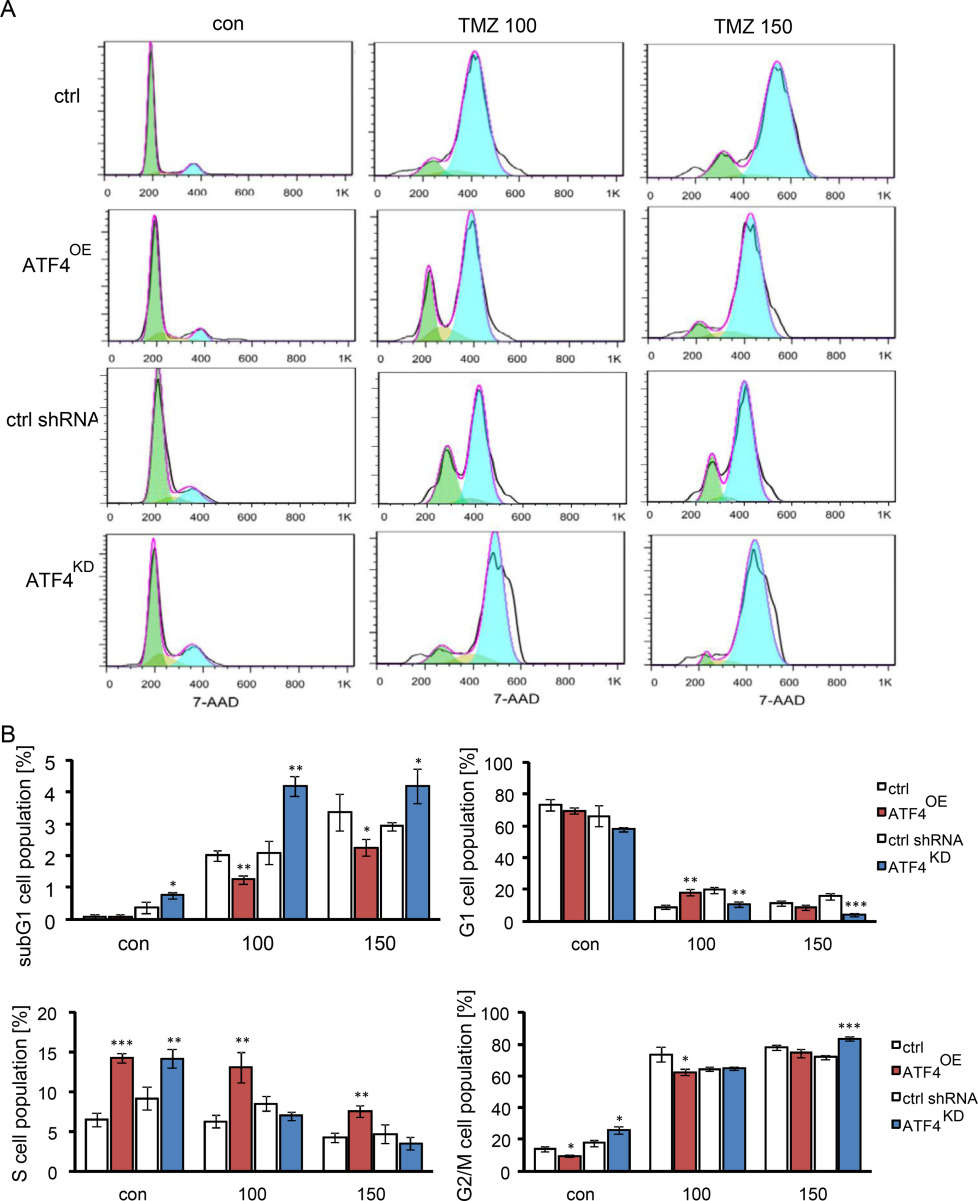
The effect of ATF4 on TMZ-induced G2/M arrest in gliomas (**A**) Cells were exposed to 100 and 150 μM TMZ for 3 days. Cell cycle profiling was conducted by flow cytometer analysis. (**B**) The graph display the percentage of G1/0, S, and G2/M DNA content analyzed from (A). Statistical analysis was performed by unpaired Student's test, *n* = 3 per group, *P < 0.05, **P < 0.01, ***P < 0.001, ctrl (peGFP-N1) versus ATF4-GFP or ATF4 shRNA versus ctrl shRNA.

To analyze the TMZ-induced cell death in glioma cells, we monitored the cell cycle profile in ATF4^OE^ and ATF4^KD^ cells using 7-AAD (Figure 4). Noteworthy, 100 μM TMZ induced increased G2/M arrest in glioma cells in a concentration-dependent manner after 3 days of treatment. The cytometric profile also confirmed that ATF4^OE^ cells show a greater cell population in S-phase and reduced numbers of cells in the sub G0-phase and G2/M-phase in comparison with controls and ATF4^KD^ cells (Figure 4A, 4B).

### ATF4 promotes glioma cell migration

**Figure 5:**
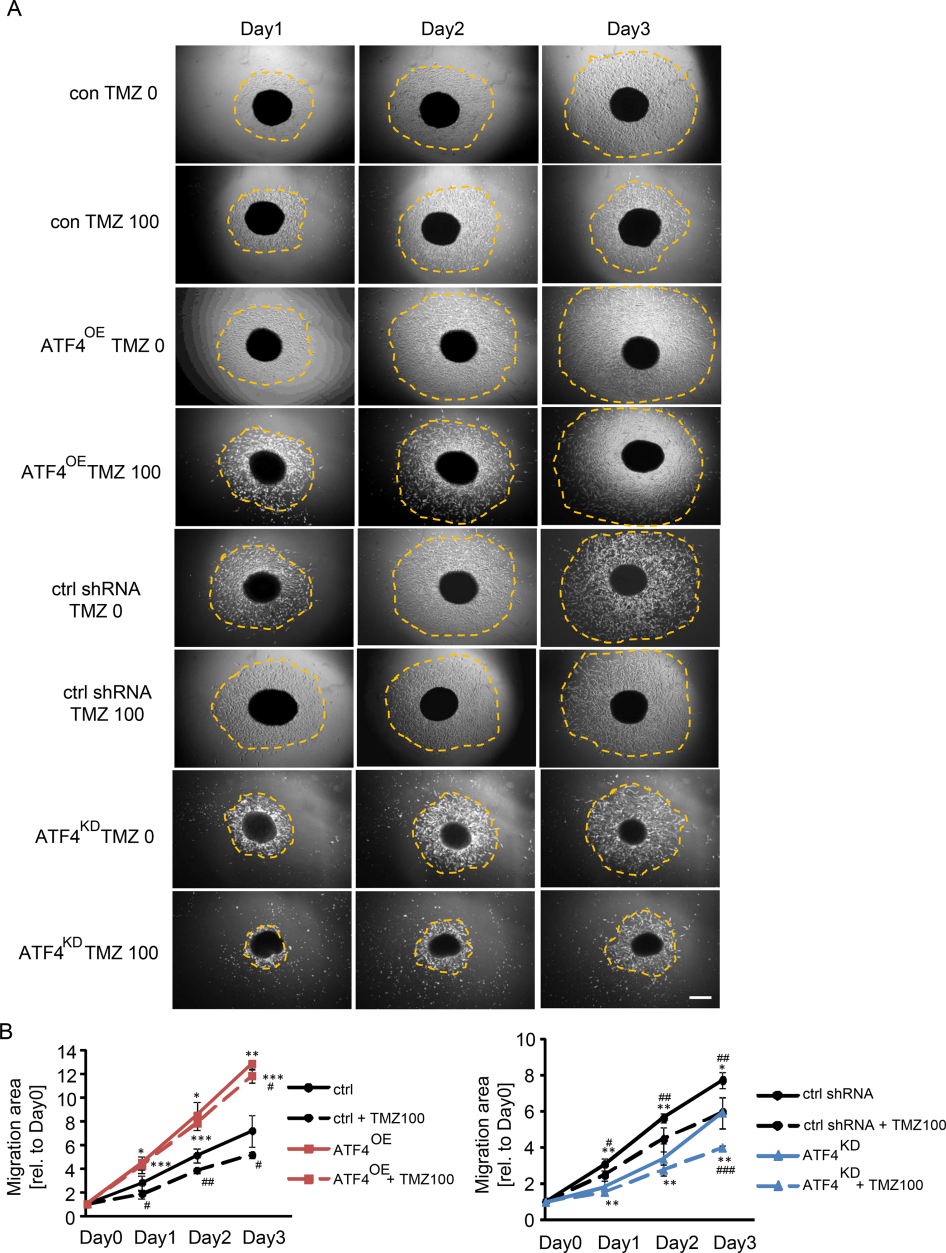
ATF4 regulates glioma cell migration (**A**) Glioma cells were cultured in a 3D sphenoid formation matrix and subjected to 100 μM TMZ for 1–3 days. The spheroid migration area was quantitatively determined (orange dotted area). (**B**) Quantification of glioma cell migration. Area measurements on day 1 to day 3 were related to the initial migration area on day 0. Scale bar represents 200 μm. Statistical analysis was performed by unpaired Student's test with *n* ≥ 3 per group. **P < 0.01, ***P < 0.001, ctrl versus ATF4-GFP or ctrl shRNA versus ATF4 shRNA. ^##^*P* < 0.01, ^###^*P* < 0.001, compared with in the presence of 100 μM TMZ versus absence of TMZ treatment.

As we demonstrated that TMZ adversely affects glioma cell proliferation and survival, we further explored the contribution of ATF4 to migration and invasion under control conditions and after TMZ treatment. Therefore, glioma cells were cultured in a 3D sphenoid formation matrix and treated with 100 μM of TMZ for 1, 2, and 3 days (Figure 5A, 5B). Following 100 μM TMZ treatment, ATF4^OE^ cells migrated significantly faster than control cells as well as ATF4^KD^ cells (Figure 5). Noteworthy, the observed effects were most prominent on day 3 (Figure 5B). Conversely, ATF4^KD^ cells showed slow migration activity when compared to controls without TMZ treatment (Figure 5A, 5B). Furthermore, glioma cell mobility was significantly suppressed following TMZ treatment (Figure 5). Altogether, these results evidence that ATF4 is a potent promoter for glioma proliferation and glioma cell motility.

### ATF4 modulates the amino acids secretion of gliomas

**Figure 6:**
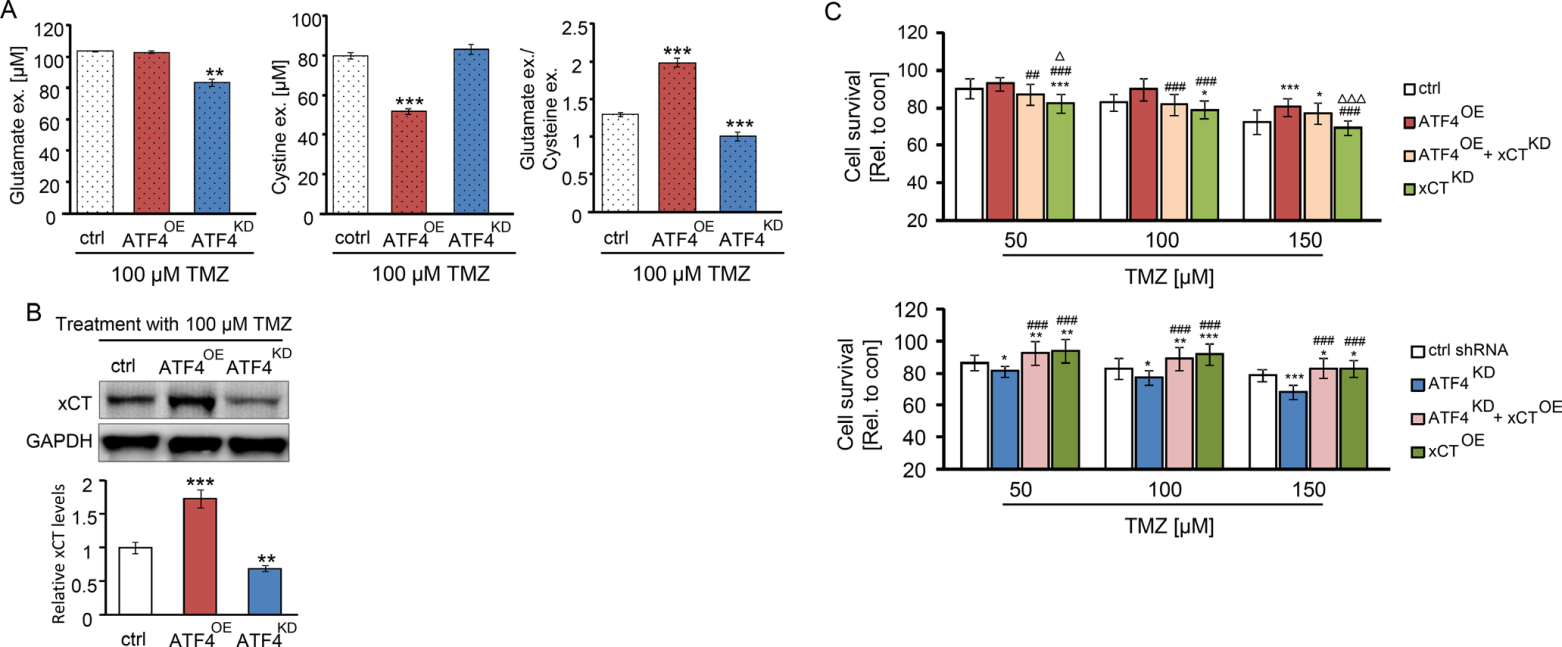
ATF4 modulates the amino acids secretion of glioma cells (**A**) Extracellular glutamate and cystine levels were evaluated by automized amino acid s analyzer. **P < 0.01, ***P < 0.001, Student's *t-test*; *n* = 3 per group. (**B**) ATF4 modulated U87 cells were treated with 100 μM TMZ for 3 days. Band densities of xCT immunoblots were normalized to the corresponding GAPDH density. (**C**) The ATF4^OE^ overexpression cells were transfected with xCT shRNA, and ATF4^KD^ knockdown cells were transfected with xCT cDNA. Cells in 96-well plates were treated with the indicated concentration of TMZ for 3 days and survival measured by MTT assay. Statistical analysis was performed by one-way ANOVA followed by LSD test where appropriate with *n* ≥ 8 per group, *P < 0.05, **P < 0.01, ***P < 0.001, compared with ctrl (peGFP-N1) or ctrl shRNA. ^#^*P* < 0.05, ^##^*P* < 0.01, ^###^*P* < 0.001 compared with cells transfected with ATF4^OE^ or ATF4^KD^, Δ*P* < 0.05, ΔΔ*P* < 0.01, ^ΔΔΔ^*P* < 0.001 compared with cells transfected with ATF4^OE^ + xCT^KD^ or ATF4^KD^ + xCT^OE^.

To identify the role of xCT in the ATF4-triggered resistance to TMZ, we evaluated the secretome of glioma cells for extracellular glutamate and cystine levels. Upon administration of 100 μM TMZ, glutamate release was significantly reduced in ATF4^KD^ U87 cells (Figure 6A). In contrast, extracellular glutamate levels were almolst equal between ATF4^OE^ U87 cells and controls (Figure 6A). Similarly, ATF4^OE^ U87 cells maintained higher levels of cystine uptake (lower levels of extracellular cystine) in comparison to controls as well as ATF4^KD^ cells (Figure 6A, middle panel). In addition, TMZ-inducible xCT transporter activity in ATF4 knockdown cells was significantly reduced (Figure 6A, right panel).

In fact, cystine is a key source for glutathione synthesis. Depletion of ATF4 has been shown to cause low levels of glutathione synthesis [24], whereas increased levels of glutathione and consequently a high ROS level has been shown to be involved in drug resistance. We found evidence for these results since xCT expression levels dropped in glioma cells after TMZ treatment (Figure 6B). Western blot analysis indicated that xCT levels were higher in ATF4^OE^ U87 cells than that in ATF4^KD^ U87 cells as well as in controls (Figure 6B).

**Figure 7:**
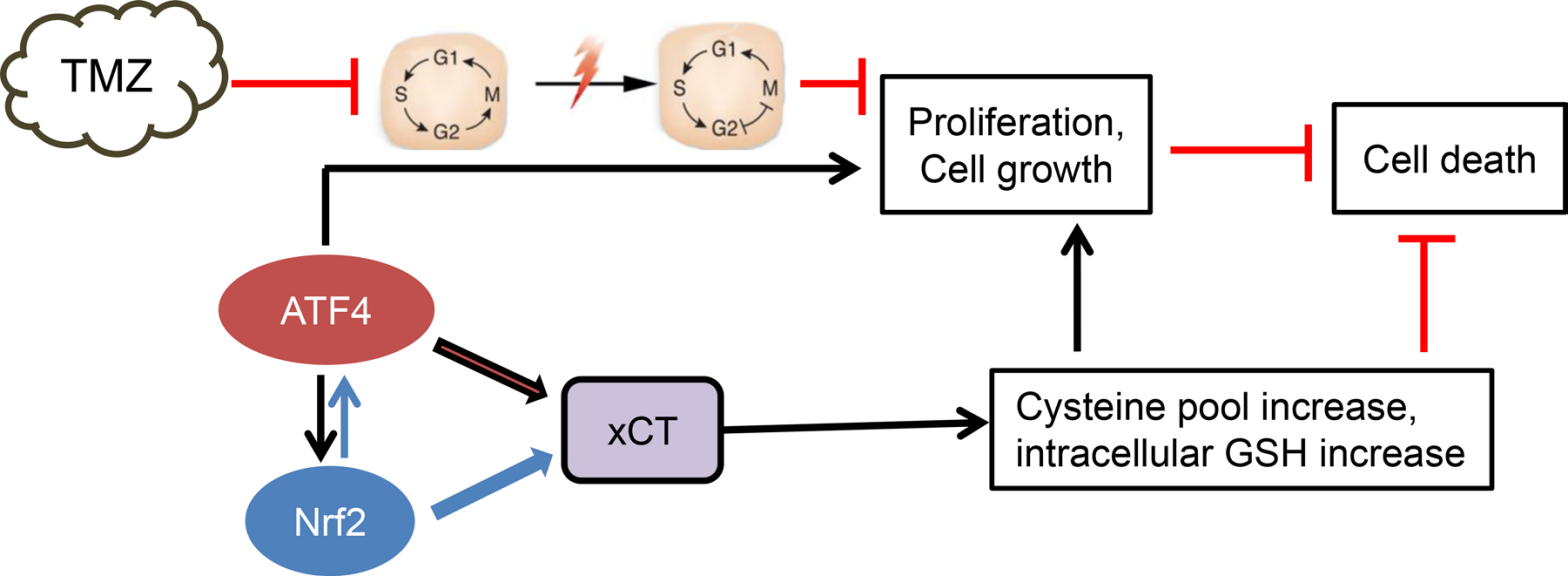
Proposed scheme of ATF4 action in chemo-resistance Our data demonstrate that ATF4 counteracts chemotherapeutic agent temozolomide (TMZ). At this point, there is evidence that ATF4 drives chemo-resistance through upregulation of xCT with subsequent elevation of glutathione. Alternatively, ATF4 regulates other genes involved in blocking TMZ effects.

The elevated levels of xCT in ATF4^OE^ cells as given in Figure 6B, prompted us to determine whether xCT causally linked with chemo-resistance in gliomas. Noteworthy, xCT expression in ATF4^KD^ cells significantly reversed the effects of TMZ-induced cytotoxicity and made these cells more chemo-resistant (Figure 6C). Conversely, knockdown of xCT mRNA partially increased the sensitivity of the ATF4^OE^ cells to TMZ-induced cell death (Figure 6C). Thus, our data demonstrate that ATF4 is involved in the regulation of xCT and this pathway modulates TMZ resistance in gliomas. (Figure 7).

## DISCUSSION

Here we investigated the role of the transcription factor ATF4 in TMZ-induced cell death. ATF4 as a oxido-metabolic driver targeting xCT is an auspicious clinically relevant drug target, as xCT is crucial for glutathione homeostasis and cell survival [2, 25, 26]. In this study, we demonstrate that ATF4 is efficient in counteracting the anti-cancer drug TMZ, leading to chemoresistance. We found that ATF4 overexpressing tumors are in particular resistant to state-of-the-art multimodal chemotherapeutics for gliomas.

Here, we hypothesized that ATF4 inhibition, although not fully lethal for glioma cells, can weaken the cellular resistance mechanisms against TMZ. The rationale for this assumption is based on the essential function of xCT in glutathione homeostasis.

First, xCT is central to the cellular cystine import in exchange to glutamate export which becomes reduced to cysteine and is mainly required for glutathione production [2, 27]. Thus, xCT is at the center stage for glutathione-dependent redox regulation and glutamate homeostasis. Second, xCT is the main glutamate exchanger in brain cancer cells thereby creating a glutamate-rich neurotoxic microenvironment [12]. Interestingly, other glutamate transporters such as EAAT1 and EAAT2 are silenced in brain cancer and high abundant system Xc- (xCT) activity results in a net balance shift towards extracellular glutamate release. Increased glutamate levels are thought to be central in advantages of glioma growth progression and invasion. Inhibition of glutamate release via ATF4 inhibition in fact disturbs tumor proliferation. Conversely, xCT overexpression can override the effects of ATF4-silencing, indicating its downstream key position in this signalling scenario.

Our data have been confirmed in independent studies: It has been demonstrated in various cancer types including malignant gliomas that ATF4 is a valid anti-cancer target. First, the redox master regulator ATF4 is abundantly expressed in glioblastoma specimens and cell lines [28]. Second, inhibition of ATF4 can induce cancer cell death in some cancer cells such as osteosarcoma, lymphomas and epithel cell derived tumors [17, 20, 29, 30].

On the other side TMZ-based chemotherapy is currently standard drug in brain tumor therapy [31, 32]. TMZ is conceptually used as a cytotoxic agent in an uni- or multimodal therapy scheme [31, 32]. Further, TMZ provides a survival benefit in a subset of patients with high grade gliomas and provides the primarily palliative treatment for the vast majority of patients. However, the increase in median survival of newly diagnosed glioblastomas treated with TMZ and radiotherapy is only 2.5 months compared with radiotherapy alone [1]. In addition, approximately one of five patients treated with TMZ develops clinically significant toxicity or acquired chemo-resistance, which can leave further treatment unsafe [33]. This situation indicates that TMZ is only a modestly effective chemotherapy calling for additional strategies. In line with this situation it would be the multicytotoxic strategy using ATF4 and xCT inhibitors for increasing the efficacy of already established standard chemotherapeutic agents. One such strategy could mean to target specifically ATF4.

There is evidence that TMZ actions are independent of the glutathione homeostasis and cystine/cysteine redox status [6]. Also, the TMZ-driven mechanisms of cell death are independent of ferroptosis and recent studies indicate that TMZ induces autophagy in tumor cells [6, 34]. However, somehow unexpected are the findings that TMZ efficacy can be supported by ferroptosis inducers [35]. ATF4 is an oxido-metabolic driver and regulator of xCT making this transcription factor a prime target. Here, we provide good evidence that ATF4 inhibition is a valid strategy overcoming chemo-resistance.

## MATERIALS AND METHODS

### Cell culture

Human U87 and U251 malignant glioblastoma multiforme cells were obtained from ATCC/LGC-2397 (Wesel, Germany). Cells were cultured in Dulbecco's modified Eagle medium (Biochrom, Berlin, Germany) containing 10% fetal bovine serum (Biochrom, Berlin, Germany) with 100 U/ml penicillin, 100 μg/ml streptomycin (Biochrom, Berlin, Germany) and 1% L-glutamine (Gibco/Invitrogen, California, USA). Cells were cultured at 37°C with 5% CO_2_ and saturated humidify.

### Chemicals

Temozolomide (TMZ) was purchased from Sigma-Aldrich (Taufkirchen, Germany). Temozolomid was solved under sterile conditions in dimethylsulphoxide (DMSO) at a concentration of 300 mM. 7-Aminoactinomycin (7-AAD) was purchased from Life Technologies (Darmstadt, Germany). Annexin V/7AAD Apoptosis Detection kit was purchased from BD Biosciences (Heidelberg, Germany). Propidium Iodide (PI) and RNAase were purchased from Sigma-Aldrich (Taufkirchen, Germany). Rotifect was obtained from Roth (Karlsruhe, Germany). Goat polyclonal to xCT antibody was purchased from Santa Cruz (Heidelberg, Germany). Rabbit polyclonal to LC3, mouse monoclonal to GAPDH antibody and Alexa Fluor^®^ IgG secondary antibody was purchased from abcam (Cambridge, UK). Polyclonal rabbit to phalloidin and 7-Aminoactinomycin (7-AAD) was purchased from Life Technologies (Darmstadt, Germany).

### Construction of plasmids carrying genes of the target proteins

The full-length complementary DNA (cDNAs) of ATF4 (human ATF4 GenBank accession no. NM_001675.4) was amplified from DNA templates (pRK7_ATF4 vector) using the following primer pairs: oligo sequences for the ATF4 construct are sense 5′-GAA GAT CTG ATG ACC GAG ATG AGC TTC CTG-3 and antisense 5-CCG GAA TTC GGA ACT CTC TTC TTC CCC CT-3. These PCR products were cloned and ligated into the BgI II-EcoR I site of the peGFP-N1 vector (Takara, Heidelberg, Germany).

### Construction of shRNA plasmids

To minimize the off-target effects of RNAi, three 19-mer short interfering RNAs were chosen for targeting the human ATF4 gene according to the critera of Ui-Tei et al. The selected ATF4 shRNAs and scrambled sequences with no homology to any known human genes were synthesized and ligated into the Bgl II/Hind III sites of pSuperGFP-neo plasmid (OligoEngine, Seattle, Washington, USA) according to the manufacture's instruction.

### Cell transfection

U87 and U251 cells were transfected with ATF4-peGFP-N1 construct, ATF4 shRNAs expression constructs and empty vectors (control, crtls) using Lipofection with Roti-Fect (Roth) according to the manufacture's instruction. After 5 days, the culture medium was refreshed containing 700 μg/ml Geneticin sulfate 418 (G418; Sigma, St.Louis, USA) for antibiotic selection. After three weeks of culturing with selected antibiotics, survival-transfected cells were collected. Stabled-transfected cells were maintained in 500 μg/ml Geneticin sulfate 418.

### Cell viability assays

Cells were seeded onto 96-well plates (8000 cells/100 μl per well) and were allowed to attach overnight. Then cells were treated with 50,100 and 150 μM TMZ for 3 days. Cell viability was assessed using 3-(4,5-dimethylthiazol-2-yl)-2,5-diphenyl- tetrazolium-bromide (MTT, 5 mg/ml in phosphate-buffered saline; Roth, Karlsruhe, Germany) for 2–3 h. Afterwards, the formazan crystals were lysed with 100 μl acidic isopropanol and the absorbance of the obtained solutions was determined at 570 nm using TECAN F50 (Crailsheim, Germany) software.

### Real time PCR analysis

Cells were collected and re-suspended in 200 μl PBS. Total RNA out of cells was extracted using High Pure RNA Isolation Kit (Roche, Mannheim, Germany) following the manual's instruction. RNA concentration was determined by NanoVue™ Plus Spectrophotometer (GE Healthcare, UK). cDNA synthesis was performed with the cDNA DyNAmo Kit ((Thermo Scientific, Germany). RT-PCR was performed with the SYBR Green PCR master mix (Thermo Scientific, Germany). Primers for ATF4, sense 5-GGT TCT CCA GCG ACA AGG-3, antisense 5-TCT CCA ACA TCC AAT CTG TCC-3; Nrf2: sense 5-TCT GAC TCC GGC ATT TCA CT-3, antisense 5-GGC ACT ATC TAG CTC TTC CA-3; xCT, sense 5-CCC AGA TAT GCA TCG TCC TT-3, antisense 5-GCA ACC ATG AAG AGG CAT G T-3; GAPDH, sense 5-GAA GG TGA AGG TCG GAG TCA-3, antisense 5-TGG AAG ATG GTG ATG GGA TT-3. ATF4, xCT and GAPDH primers for RT-PCR were purchased from metabion international AG (Germany). Real time cycling parameters: initial activation step (95°C, 15 min), cycling step (denaturation 94°C, 15 s; annealing at 56°C or 60°C, 30 s; and finally extension for 72°C, 30 s X40 cycles), followed by a melting curve analysis to confirm specificity of the PCR using Light Cycler 480 (Roche, Germany). The Ct value was corrected by Ct reading of corresponding GAPDH as control.

### Western blotting

Cells were lysed with NP40 buffer containing 5 mM NaF and a protease inhibitor cocktail (Roche, Basel, Switzerland). After quantification with the NanoVue™ Plus Spectrophotometer (GE Healthcare, UK), samples were mixed with 4× loading buffer and 10× NuPAGE^®^ Sample Reducing Agent (Invitrogen, California, USA). Equal amounts of protein lysates were loaded and separated by 10–12% SDS-NuPage gel (Invitrogen, CA, USA) and electrophoresis was performed in MOPS-buffer, transferred to polyvinylidene difluoride (PVDF) membranes (Roth, Karlsruhe, Germany). Membranes were blocked in Tris-buffered saline (pH7.4) with 1% Tween-20 (TBST) with 2% Magic block and 10% Top block (Lubio science, Luzern, Switzerland) for 20 min, and then hybridized with antibodies against xCT (1:1000) and GAPDH (1:2000) in 5% BSA-TBST overnight at 4°C. Following 3× for 5 min washes in TBST, the membrane was incubated with HRP-conjugated secondary antibodies (1:3000) at room temperature for 1 h. Detection was performed with ECL plus kit (GE-healthcare, Solingen, Germany).

### Immunofluorescence staining

A total of 1 × 10^5^ cells/well were seeded onto sterile glass coverslips. After 72 h treatment with 100 μΜ TMZ, cells were fixed using 4% paraformaldehyde (PFA), and then incubated with a rabbit polyclonal anti-LC3 (1:500) antibody overnight. Cells were washed twice with PBS, incubated for an additional 1 h with Alexa Fluor^®^647 anti-rabbit IgG antibody (1:1,000), then stained with phalloidin (1:500) and counterstained Hoechst 33258 (1:10,000). Coverslips were mounted on slides with Immu-Mount (Thermo scientific, Massachusetts, USA). Images were taken by an Axio Observer with the Zen Software (Zeiss, Oberkochen, Germany).

### Three dimensional spheroid migration assay

100,000 cells per spheroid were packaged in 100 μl mixture with 32% methylcellulose solution and 68% culture medium. Cell packages were dropped on the petri dishes and reversely cultured under standard humidified conditions (37°C, 5% CO_2_) for 12 h so that the package is stereoscopic with all the cells aggregate to the top and formed spheroid. Afterwards the spheroid was moved to the 48-well plate and kept culturing with medium. After 12 hours, the spheroid attached to the bottom of the well and the cells migrate, and the pictures of spheroids were taking on the time points day 1, day 2, day 4 and day 6 by Olympus IX71 microscope, and the cells growth area of the spheroid were measured by Image J software.

### Cell death analysis by 7-AAD and annexin V assay

Glioma cells were treated with TMZ at indicated concentration for 3 days, and then harvested by trypsinization and pelleted by centrifugation. Cells were re-suspended in 100 μl Annexin binding buffer with 5 μl FITC Annexin V and 5 μl 7-AAD (BD Biosciences, Germany) for 15 min at room temperature in the dark. After incubation, cells were transferred to FACS tubes and analyzed by Flow Cytometer BD FACSCanto II (BD Bioscience, Heidelberg, Germany). A minimum of 10,000 cells were counted and analyzed per condition. Analyses were carried out with the FlowJo Software 7.6 (Ashland, Oregon, USA).

### Cell cycle analysis

200,000 glioma cells/well were seeded overnight in 6-well dishes (Corning Life Sciences, Germany). The next day, cells were treated with TMZ for 72 h. Cells and media supernatant were collected, washed with PBS and then fixed with 70% ethanol solution overnight at 4°C. Fixed cells were washed twice with PBS solution and afterwards resuspended in Hypotonic lysis buffer (0.1% sodium citrate, 0.1% Triton X100, 100 μg/ml RNAse) for 20 min. Cell cycle analyses were performed within 2 h after adding 7-AAD (7-aminoactinomycin D, Molecular Probes, Invitrogen, Darmstadt, Germany) by Flow Cytometer BD FACSCanto II (BD Bioscience, Heidelberg, Germany). Analyses were carried out with FlowJo Software 7.6. A minimum of 10,000 cells were counted and analyzed per condition.

### Metabolic assays

The release of amino acids from glioma cells into the extracellular medium were detected using high performance liquid chromatography (HPLC). For TMZ treatment experiments, 400,000 cells/well were seeded overnight into 6-well dishes (Corning). The next day, cells were incubated in glutamine-free DMEM (Biochrom AG, Berlin, Germany/Sigma) with indicated concentration of TMZ for 24 h. The supernatants were collected and measured by using. Amino acids were analyzed by ion-exchange chromatography and post-column ninhydrin derivatization technique using a fully automated amino acids analyzer (Biochrom 30+, Laborservice Onken, Gründau, Germany). For the amino acid analysis, 100 μl of sample was deproteinised with 100 μl of 10% sulphosalicylicacids. Afterwards, 20 μl of this supernatant was then loaded by the autosampler into a cation-exchange resin-filled column.

### Statistical analysis

Data are expressed as mean ± s.d. taken from at least three independent experiments, and compared with two-sided unpaired Student *t-test* or Mann-Whitney test where appropriate. One-way ANOVA was used for comparisons of more than two groups. When the ANOVA was significant, *post hoc* testing of differences between groups was performed using LSD test. A *P-value* < 0.05 was considered statistically significant.
